# Opportunities for Increased Physical Activity in the Workplace: the Walking Meeting (WaM) Pilot Study, Miami, 2015

**DOI:** 10.5888/pcd13.160111

**Published:** 2016-06-23

**Authors:** Hannah E. Kling, Xuan Yang, Sarah E. Messiah, Kristopher L. Arheart, Debi Brannan, Alberto J. Caban-Martinez

**Affiliations:** Author Affiliations: Hannah E. Kling, Xuan Yang, Sarah E. Messiah, Kristopher L. Arheart, Miller School of Medicine, University of Miami, Miami, Florida; Debi Brannan, Western Oregon University, Monmouth, Oregon.

## Abstract

**Introduction:**

Despite the positive impact walking has on human health, few opportunities exist for workers with largely sedentary jobs to increase physical activity while at work. The objective of this pilot study was to examine the implementation, feasibility, and acceptability of using a Walking Meeting (WaM) protocol to increase the level of work-related physical activity among a group of sedentary white-collar workers.

**Methods:**

White-collar workers at a large university were invited to participate in a newly developed WaM protocol. Workers who conducted weekly meetings in groups of 2 or 3 individuals were recruited for the pilot study (n = 18) that took place from January 2015 to August 2015. Seventeen participants wore an accelerometer to measure physical activity levels during 3 consecutive weeks (first week baseline, followed by 2 weeks of organized WaMs) and participated in focus groups conducted during week 3 to document experiences with the WaM protocol.

**Results:**

The WaM protocol met study criteria on feasibility, implementation, and acceptability among study participants. The average number of minutes (standard deviation) participants engaged in combined work-related moderate/vigorous physical activity per week during the 3 weeks increased from an average of 107 (55) minutes during the baseline week to 114 (67) minutes at week 2 and to 117 (65) minutes at week 3.

**Conclusion:**

White- collar workers were supportive of transforming regular seated meetings into walking meetings and increased their work-related physical activity levels.

## Introduction

Prolonged periods of time sitting may increase risk of premature death, have associations with health problems, and compromise metabolic health (1). Walking decreases risk for all-cause mortality because of its positive impact on physical health (2). Engaging in moderate exercise, which includes brisk walking, for as little as 15 minutes per day can add up to 3 years of life expectancy (3). The American Heart Association recommends walking for at least 30 minutes per day to reduce the risk for chronic diseases such as osteoporosis, breast and colon cancer, and type 2 diabetes (4). The Centers for Disease Control and Prevention (CDC) recommends walking at least 10,000 steps per day (5). A recent study documented that 3 brief sessions of higher-intensity physical activity (PA) of at least 10 minutes throughout the day has benefits for physical health (6). Despite the positive impact walking has on human health, few opportunities exist for workers with largely sedentary jobs to increase PA while at work.

Professional white-collar work environments typically do not support PA throughout the workday (7); blue-collar workers report greater physical demands than do white-collar workers (8,9). Finding ways to incorporate PA into white-collar work settings could benefit many workers. One way to incorporate and encourage PA is by walking during a meeting instead of sitting. No studies have yet evaluated the use of walking meetings in the workplace. A recent Cochrane review examining interventions to reduce sitting in the workplace noted that no studies yet have investigated the effect of periodic breaks or standing or walking meetings (10). The objective of our pilot study was to examine the acceptability among white-collar workers of engaging in walking meetings during the workday and the feasibility of organizing these meetings around their work schedules. We also examined the implementation of the Walking Meeting (WaM) protocol and assessed the impact of the protocol on work-related PA levels in a sample of white-collar workers.

## Methods

A sequential explanatory mixed-methods study design was implemented to develop the WaM protocol and then assess the feasibility, acceptability, and implementation of the protocol among a sample of white-collar workers during an 8-month period (January 2015–August 2015) at a university in an urban setting in southern Florida. We recruited by email and obtained consent from 18 university faculty members, staff leaders, and staff members (8 groups of 2 or 3 members) to participate in a 1-week baseline assessment and 2-week walking meeting protocol. One participant lost an accelerometer and was released from the study, so the final analytic sample was 17 participants.

Eligibility criteria for study participation included groups that had an established meeting time and met at least weekly for 30 or 60 minutes. Study participants had to be aged 18 years or older, full-time university employees, able to wear an accelerometer at the hip for 3 consecutive work weeks, able to read and write in English, not absent from work for more than 1 workday during study participation, and able and willing to walk for 30 minutes during the group’s regular meeting time. Individuals who self-reported chest pain; prior myocardial infarction; cardiopulmonary, spinal or lower limb surgery in previous 6 months; or a recent history of uncontrolled hypertension were not eligible to participate.

Accelerometers were calibrated at baseline, and the WaM protocol was created and then delivered to participants in a workshop on the first day of the baseline week. Each group was instructed to carry on with their traditional sitting meetings and regular work schedule during the first week of baseline data collection. After the baseline week, each group was asked to modify one standard sitting meeting according to the WaM protocol for 2 consecutive weeks. At the start of the study and at the end of each of the 3 weeks of participation, researchers met with each group to administer a survey that collected self-reported PA measures. At the end of both walking meeting implementation weeks, accelerometers were collected, the last survey was administered, and a focus group meeting was conducted with each walking group. The WaM pilot study was approved by the institutional review board of the University of Miami.

### Development of the WaM protocol

We developed a 7-item core component walking meeting protocol that included a safe 25- to 30-minute walking path on the university campus. To develop the WaM protocol, we conducted formative research on traditional meeting protocols. For example, every traditional meeting has an agenda, an objective, and someone who directs the meeting. Next, we reviewed research on how walking meetings are conducted. The mainstream literature outlined a common approach to conducting a walking meeting, and the grey literature provided anecdotal tips and instructions that suggested a similar setup and design (ie, create an agenda, assign roles, take notes, and so on) (10–13). From this formative research on traditional and walking meetings (12–14), common themes were combined and outlined into a 7-item core component list that reflects best practices for walking meetings and serves as the foundation for a uniform WaM protocol (Box). The WaM protocol was assessed across a 3-week period: the first week was a baseline observation period for each participant’s typical work-related PA levels, and the subsequent 2 weeks were observation periods for each participant’s walking meeting–related PA levels.

**Box. Core Components of the Walking Meeting Protocol in the Walking Meeting (WaM) Pilot Study**

- Set a time and place to meet before your WaM.
- Create an agenda for your WaM.
- To make the walk more comfortable, bring items such as water, sunglasses, and sunscreen. Wear comfortable shoes.
- Have the group leader assign roles to each walking meeting group member. (ie, time checker, note taker, path leader).
- Follow the prescribed route.
- Walk for at least 30 minutes.
- After the walking meeting, sit and conclude to wrap up meeting; take care of paperwork or other tasks that could not be accomplished during WaM.

### Study measures (survey and accelerometry)

After recruitment and consent, study participants were asked at baseline to complete a paper-based survey with questions on sociodemographic characteristics and standardized measures of self-reported PA (the International Physical Activity Questionnaire [IPAQ]) (15). Although we administered the entire IPAQ to study participants, for this analysis we used only the 6 work-related PA questions. The identical survey measures were administered 3 more times: during the first week of the walking meeting, during the second week of the walking meeting, and during the focus groups. Participants were asked at baseline to wear a GT3XP Actigraph accelerometer (ActiGraph, LLC) on their waist for 21 consecutive days. On the first day, the accelerometer was initialized by the research team and participants were shown how to attach and remove the device. Participants were instructed to wear the accelerometer all day except while they were sleeping, taking a shower, or swimming. Participants also kept a daily log throughout the 3 weeks of the pilot study documenting the beginning and end of work shifts along with times they wore the accelerometer and when they removed it. The accelerometers recorded data in 1-minute intervals, providing the number of counts for each minute for 21 days. A count was defined as any activity that was measured by the accelerometer. At the end of 21 days, the researchers retrieved the accelerometer from the worker and downloaded its data to the study computer.

Accelerometer data were parsed by using ActiLife software version 6 (ActiGraph, LLC) into 2 data sets: a set of minutes associated with the workday and a set of minutes associated with the non-workday. For minutes associated with the workday, each minute was assigned a level of PA intensity according to definitions established by Freedson and colleagues (16): light (101–1,952 counts per minute), moderate (1,953–5,724 counts per minute), vigorous (5,725–9,498 counts per minute), and very vigorous (≥ 9,499 counts per minute). PA levels were analyzed on the day of the week each group had its walking meeting. For example, if a group held its walking meeting on Wednesday during week 2 and week 3, we collected data on the number of steps taken on those implementation days and on the previous Wednesday of their baseline week.

Measures of WaM protocol implementation, feasibility, and acceptability were defined (Table 1). Focus group sessions were conducted to ask participants questions about the extent to which the WaM protocol was implemented, whether the WaM protocol was feasible, and whether it was acceptable to study participants.

**Table 1:** Metrics Used to Assess Feasibility, Acceptability, and Implementation of the Walking Meeting (WaM) Pilot Study, 2015

Objective	Key Questions	Data Source	Indicators
Feasibility	Are white-collar workers willing to consider adopting walking meetings?	Response rate	Number of eligible employers who agree to participate
	Can research teams and their members be trained to deliver the walking meeting strategy?	Interviews with teams involved in walking meeting training	Percentage of teams and team members that complete both walking meetings (week 2 and week 3)
	How much and what types of support are needed by worksite staff to enact walking meetings?	Interviews with WaM team members	Time spent by research team members providing technical assistance and other support
Acceptability	How do those involved with walking meetings view the strategy?	Interviews with WaM team members	Percentage of respondents who report that the walking meetings strategy is acceptable
	To what extent is the walking meetings strategy viewed as suitable for the setting and population?	Interviews with WaM team members	Percentage of respondents who report that the strategy is suitable for their setting
	Can the walking meeting strategy be adapted to suit research team leadership and membership needs and preferences?	Interviews with WaM team members	Recommendations and preferences communicated
Implementation	To what degree are core components of the walking meeting strategy implemented?	Team meeting discussion plan and process tracking	Integrated worksite policy is written and communicated; cost and resources expended.
	What noncore or adaptive elements are implemented?	Team meeting discussion plan and process tracking	Number and types of trainings and information delivered
	To what extent is walking meeting strategy implemented with fidelity?	Interviews with WaM team members	Percentage of participants that implemented the suggested components of a walking meeting

### Focus groups

To complement our survey data, we conducted a 1-hour focus group with each walking group at the end of the pilot study to discuss 3 broad domains: working experience and job organization, organizing the walking meeting, and conducting the walking meetings. The focus group team consisted of a moderator and another researcher who collected paperwork and took notes. The focus group questions were developed after collecting the quantitative data and were based on the WaM protocol. We used grounded-theory methodology (17,18) to develop a discussion guide, which consisted of a mix of 4 to 7 open-ended questions for each domain. 

### Statistical analysis

To examine how the walking meetings affected objectively measured PA, we compared accelerometry data for the day of the baseline week with the accelerometry data for the 2 days of the walking meetings. Four levels of PA were examined: light, moderate, vigorous, and very vigorous. CDC’s guidelines for adequate PA suggest that aerobic activity of moderate intensity or higher (vigorous or very vigorous) is important for health benefits (19). Therefore, we combined moderate, vigorous, and very vigorous PA levels into one category to examine changes in PA across the 3-week study period. Using STATA version 13.0 (StataCorp LP), we calculated mean (standard deviation [SD]) number of minutes spent during the workday for each walking meeting day and the corresponding baseline day. Differences between periods were assessed by using paired *t* tests. We used conservative 2-tail tests and emphasize that this descriptive study was not designed to determine cause and effect.

Focus group discussions were transcribed, and text was entered into NVivo version 10 (QSR International Pty Ltd) for electronic coding, data retrieval, and analysis. Inductive thematic analysis was used to identify main themes across groups. Emerging themes were verified through discussion, and a coding framework based on the themes and domains was developed (20). Transcripts were coded by H.E.K. and reviewed by the study team. The coding was reviewed by 2 coauthors (A.J.C-M. and X.Y.); existing codes were developed and new codes were identified.

## Results

The 17 participants had a mean age of 39.8 years (SD, 12.2 y); 4 were men and 13 were women; 4 were white and 13 of another race; 12 self-identified as Hispanic. Mean body mass index was 25.8 kg/m^2^. Two participants completed some college or technical school, 9 were college graduates, and 6 had a master’s degree or higher. Seven team leaders who were invited to participate expressed interest in the study but did not meet eligibility requirements. The overall response rate was 53% (8/15). 

Initially, the sample comprised 8 team leaders, 2 groups of 3 members, and 6 groups of 2 members; one participant eventually dropped out. Of the 8 groups, all completed at least one walking meeting and 7 groups completed both walking meetings. Taken together, the study enrollment, response rate (17/18 participants, or 94%), and completion of walking meetings (7/8 groups, or 88%) suggest that walking meetings were feasible for this sample of white-collar workers.

All participants agreed to the WaM strategy after being taught how to organize and conduct the walking meeting during study initiation. Although the protocol suggested 30 minutes as a minimum for the walking meeting, all groups walked from 30 to 40 minutes. Five groups followed the prescribed path. Of the 3 groups that did not follow the path, 2 groups took a different route to accomplish tasks at other campus locations during their walking meeting; the third group took a wrong turn but later rejoined the prescribed path. All groups agreed that the WaM protocol was acceptable according to the measures defined (Table 1), including the suitability of walking meetings for their work setting.

When we asked focus group participants about the extent to which they implemented the components of the WaM protocol, 7 groups created and printed an agenda to take on their walk, 3 groups took written notes, and one group engaged in a sit-and-conclude session after their walk. All groups used their established meeting times and places for their walking meetings, and all groups felt they had proper attire and items to make their walk comfortable. Six groups completed 5 of 7 prescribed WaM protocol components. The 2 components least frequently completed were the sit-and-conclude session and creating an agenda.

The average number of minutes (standard deviation) participants engaged in combined work-related moderate/vigorous physical activity per week during the 3 weeks increased from an average of 107 (55) minutes during the baseline week to 114 (67) minutes at week 2 and to 117 (65) minutes at week 3. The mean (SD) number of minutes in light PA on walking-meeting days among all participants decreased from 169.8 (83.3) minutes at baseline to 129.2 (62.1) minutes at week 3 (Table 2); however, the differences between baseline and each follow-up week were not significant. On the day of the walking meeting, the mean number of minutes spent in moderate, vigorous, or very vigorous PA increased from 34.1 minutes at baseline to 43.5 minutes at week 2 (*P* = .31) and to 43.0 minutes at week 3 (*P* = .33 for difference from baseline). Nine participants did not meet the 10,000 steps goal during baseline week; by week 3, two of these 9 participants reached 10,000 steps.

**Table 2:** Mean (Standard Deviation) Number of Minutes of Work-Related Physical Activity^a^ on Baseline Day (Nonwalking Day) and Walking Days (Week 2 and Week 3) in the Walking Meeting (WaM) Pilot Study, 2015^b^

Level of Physical Activity	Baseline^b^ (n = 17 Participants)	Week 2^b^ (n = 17 Participants)	Week 3^b^ (n = 14 Participants)
Light	169.8 (83.3)	143.3 (46.1)	129.2 (62.1)
Moderate	31.4 (19.9)	41.6 (25.2)	40.4 (22.4)
Vigorous	1.2 (3.2)	1.83 (6.3)	1.6 (4.4)
Very vigorous	1.5 (5.3)	0.01 (0.04)	1.1 (4.0)
Moderate, vigorous, and very vigorous	34.1 (23.6)	43.5 (29.6)	43.0 (26.6)

a Work-related physical activity is all activity that took place and was measured by accelerometry between the hours of 9:00 a.m. and 5:00 p.m. on a workday.

b No significant difference (using paired *t *test) in the number of light, moderate, vigorous, or very vigorous physical activity minutes between baseline and week 2 or week 3.

When asked about their typical meeting at work, participants had the following comments: “Sometimes people come into our meeting. Sometimes our meetings get cut short. Sometimes we have to come back to our meeting” [Group 4, participant 1]. “[We] talk about personal stuff and other study-related stuff. Scheduling, vacation, coverage, etc. They (the meetings) are very informal” [Group 7, participant 1]. “Most meetings go overtime” [Group 1, participant 1].

When asked how teams organized their walking meetings, participants stated the following:

Often times, for our meetings, I know I’m going to be in front of a computer. So, I kinda multitask during our meetings. So I really had to set time for these walking meetings versus plan to be in front of my computer, talking to [another participant] and doing something else, which is rude but you know, it’s reality” [Group 4, participant 2].

“We both agreed on a time. Scheduled it on our calendar” [Group 3, participant 1]. “Made mental bullet points. Didn’t take paper with us” [Group 2, participant 1].

When asked about their general experience at engaging in walking meetings, participants stated, “Being outside with other people, you’re never really alone, per se, and you never feel as if you are this small little bubble doing some random thing, like you’re in the world and you’re doing something” [Group 5, participant 2]. “Today I used it as a way of letting go of my stress” [Group 5, participant 3].

I loved it [walking meeting]. It was very energizing, very invigorating. We got a lot done, we went through our agenda completely, efficiently, and it helped us generate ideas as we were discussing a topic. We were generating ideas for solutions and came up with solutions and tasks, like who’s going to do what. I was incredibly efficient” [Group 6, participant 3].

## Discussion

Traditional seated meetings that were converted into a walking format using the WaM protocol increased moderate, vigorous, and very vigorous PA levels by 10 minutes among our sample of white-collar workers. Many jobs in the white-collar workforce involve a disproportionate amount of sitting time, which can increase the risk for being overweight or obese (21). Although several interventions have aimed to increase PA levels in the workplace (eg, by using stability balls instead of chairs and sit–stand work stations instead of traditional desks), the scientific literature consists of either low-quality evidence or equivocal results on the effect of these interventions. Data from this pilot study suggest that walking meetings might provide an alternative to the sedentary workdays of white-collar workers.

Focus groups suggest that the WaM protocol was feasible, accepted, and successfully implemented by study participants. Among the 8 participating groups, 7 completed both walking meetings. These findings are in contrast with those of Cooper et al, who found that university employees who reported a lack of time for PA and perceived that fitness facilities at work were expensive did not engage in PA (22). We found that walking meetings were accepted and implemented by white-collar university employees in this pilot study and that these workers could easily fit a walking meeting into the workday with little to no burden to their workflow.

This study has several limitations, including a small number of participants and a short duration. Time constraints (a 7-month academic year) and a limited number of accelerometers prohibited the research team from recruiting more participants and conducting additional cycles. The use of accelerometers may have caused a Hawthorne-type or reactivity event (23): participants may have increased their PA at work because they knew they were being monitored.

Despite these limitations, this pilot study has several strengths, including the collection of both quantitative and qualitative data, a flexible walking meeting protocol, and a uniform community of workers who engaged in similar tasks. A unique aspect of this study was the flexible walking meeting protocol. Rather than allowing participants to engage in a walking meeting without guidance, the research team provided participants with suggestions and a prescribed route with the aim of eliminating confusion, providing autonomy, and stimulating productivity while walking in a safe work environment. This study adds information on walking meetings to the scientific literature, where little research exists.

The data collected from this pilot study suggest that walking meetings, a simple modification of traditional seated meetings, were not only well accepted by our sample of white-collar workers but were easy to implement and feasible to conduct during regular working hours. PA interventions such as the WaM protocol that encourage walking and raise levels of PA in the workplace are needed to counter the negative health effects of sedentary behavior. Future studies should consider more frequent and repeated measures of walking meetings with larger groups of white-collar workers.
